# Tuning the d-Band Center of Nickel Bimetallic Compounds for Glycerol Chemisorption: A Density Functional Study

**DOI:** 10.3390/molecules30030744

**Published:** 2025-02-06

**Authors:** Carlos M. Ramos-Castillo, Luis Torres-Pacheco, Lorena Álvarez-Contreras, Noé Arjona, Minerva Guerra-Balcázar

**Affiliations:** 1 Centro de Investigación y Desarrollo Tecnológico en Electroquímica S. C., Sanfandila, Pedro Escobedo, Querétaro C. P. 76703, Mexico; wvelazquez@cideteq.mx; 2 Facultad de Ingeniería, División de Investigación y Posgrado, Universidad Autónoma de Querétaro, Querétaro C. P. 76010, Mexico; 3 Centro de Investigación en Materiales Avanzados S. C., Complejo Industrial Chihuahua, Chihuahua C. P. 31136, Mexico; lorena.alvarez@cimav.edu.mx

**Keywords:** fuel cells, electro-oxidation, bimetallic compounds, d-band, DFT

## Abstract

The modification of catalytic activity through the use of metallic promoters is a key strategy for optimizing performance, as electronic factors play a crucial role in regulating catalytic behavior. This study explores the electronic factors behind the adsorption of glycerol (Gly) on bimetallic nickel-based compounds (${\mathrm{Ni}}_{3}X$) using density functional theory (DFT) calculations; incorporating Mn, Fe, Co, Cu, and Zn as promoters effectively tunes the d-band center of these systems, directly influencing their magnetic, adsorption, and catalytic properties. A good correlation between the calculated glycerol adsorption energy and the d-band filling of the studied bimetallic surfaces was identified. Interestingly, this correlation can be rationalized using the celebrated Newns–Anderson model based on the calculated d-band fillings and centers of the systems under study. Additionally, the adsorption energies and relative stability of other electro-oxidation intermediates toward dihydroxyacetone (DHA) were calculated. Notably, the ${\mathrm{Ni}}_{3}\mathrm{Co}$ and ${\mathrm{Ni}}_{3}\mathrm{Cu}$ systems exhibit an optimal balance between glycerol adsorption and DHA desorption, making them promising candidates for glycerol electro-oxidation. These theoretical insights address fundamental aspects of developing glycerol valorization processes and advancing alcohol electro-oxidation technologies in fuel cells with noble-metal-free catalysts.

## 1. Introduction

The electrochemical oxidation of alcohols presents a promising route for sustainable energy conversion and hydrogen production, with the success of this process primarily relying on catalyst performance. Historically, noble metals such as platinum (Pt), gold (Au), and palladium (Pd) have been preferred for their superior catalytic properties [1,2,3,4]. These metals are highly effective in activating alcohol molecules for oxidation, producing valuable products such as dihydroxyacetone [5]. Among them, Pt-based catalysts are widely used as model systems for methanol electro-oxidation [6,7,8]. However, the high cost and limited availability of noble metals have motivated research on more affordable and abundant alternatives, including metal oxides, transition metal alloys, and bimetallic compounds [9,10,11,12,13,14,15].

Bimetallic compounds, particularly those with the formula $A_{3}X$ based on non-noble metals, are noteworthy due to their ability to modulate catalytic activity by incorporating a secondary metal [16,17,18]. This incorporation alters the electronic properties of the active site, thereby influencing the adsorption of reaction intermediates and modifying the catalytic behavior [7,8]. The extensive number of potential bimetallic combinations derived from transition metals underscores the need for a synergistic approach combining theoretical and experimental methodologies. Experimental techniques validate plausible electro-oxidation pathways and products, offering essential insights into catalytic efficacy. Conversely, computational models assist in identifying the most promising compounds and desirable characteristics for rational catalyst design. This integration is crucial for advancing the development of efficient and sustainable catalysts for energy applications.

Recently, Santos et al. [19] proposed a computational screening model that correlates the adsorption energies of oxygen and carbon with glycerol and specific intermediates on pure transition metals, extending these trends to bimetallic compounds to identify promising candidates for enhanced catalytic performance in electrochemical reactions. Despite these advancements, many fundamental aspects remain unexplored, particularly in understanding atomic-level chemical bonding and catalyst interactions. Identifying optimal catalysts requires insight into the chemical bonds formed between adsorbates and the catalyst surface [20,21,22,23,24]. In any catalytic cycle, the process begins with reactant adsorption, followed by bond rearrangement or diffusion of the adsorbed species [25,26,27]. The bond strength between adsorbates and the catalyst is key to understanding catalytic functionality. Important questions include the role of promoters in tuning the catalyst’s electronic structure, their effect on adsorption sites, and the stabilization of reaction intermediates. Addressing these gaps is essential for the rational design of more efficient and selective catalysts.

Additionally, the magnetic properties of many transition metals used in the electrocatalysis of bimetallic systems have gained increasing importance in recent years. The d-band model must be extended to account for the effects of magnetism on catalytic activity. A magnetic moment can significantly influence the electronic structure of the catalyst and particularly the distribution of d-electrons [28,29]. This is especially relevant for transition metals such as Ni, Fe, Co, and Mn, where magnetic ordering affects the adsorption energy of key intermediates. Recent studies, including that by Cao et al. [30], have demonstrated that the spin state of magnetic metal surfaces notably impacts their chemical properties. Specifically, stronger adsorption energies are observed on non-spin-polarized surfaces compared to spin-polarized ones, a trend that is consistent across Fe, Co, and Ni. These findings highlight the relationship between surface electronic structure and adsorption behavior, which can be explained through a simplified d-band model. Moreover, the manipulation of spin effects, such as through the use of metal promoters, has been suggested as a strategy to enhance surface reactivity. Bhattacharjee et al. [31] further emphasize the necessity of incorporating spin polarization effects into the d-band model for both adsorption and certain catalytic reactions. In these scenarios, while the reactants and products may be non-magnetic, the reaction intermediates can exhibit magnetic properties, thereby making the rate-determining steps dependent on the spin exchange between the adsorbate and the metal surface.

Motivated by the desire to uncover the fundamental factors influencing the chemisorption of alcohols on bimetallic surfaces, as well as the ongoing search for new catalysts based on non-noble metals, this study focuses on understanding the electronic properties of bimetallic nickel-based compounds (${\mathrm{Ni}}_{3}$X) and their interactions with glycerol. By incorporating various third-row transition metals, from V to Zn, we aim to elucidate how these elements modify the d-band center and magnetic properties and how they impact the adsorption and catalytic behavior of the surfaces. Additionally, this study seeks to explore possible correlations between magnetic properties and adsorption energy. The ultimate goal is to identify promising candidates for glycerol electro-oxidation that can enhance the development of sustainable, noble-metal-free catalysts for fuel cell applications.

## 2. Computational Methods

**Figure 1:**
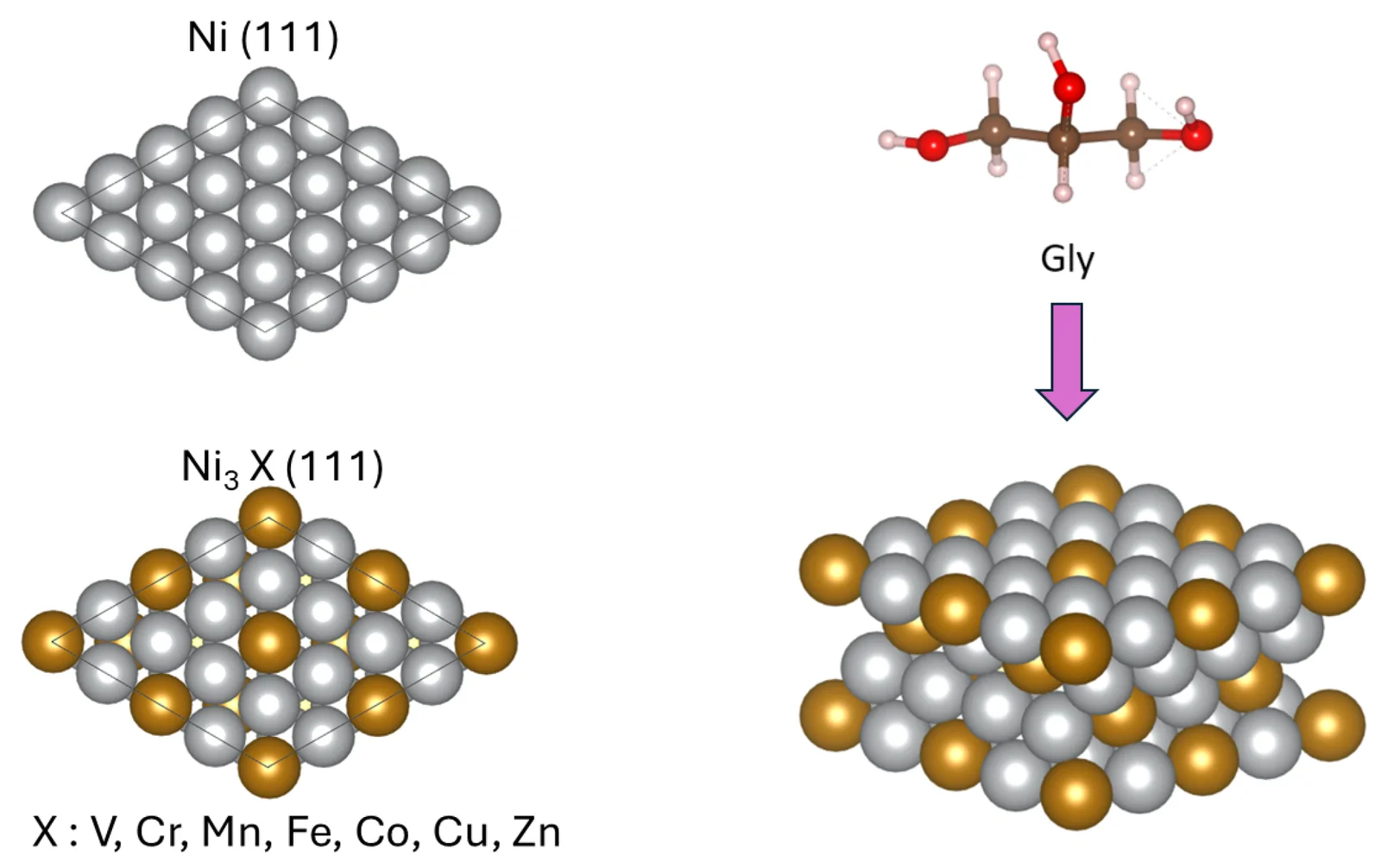
Atomic structure of Ni_3_X (111) bimetallic surfaces under study (X = V–Zn).

Total energy calculations were carried out within the framework of the projector-augmented wave (PAW) method, as implemented in the Vienna Ab initio Simulation Package (VASP) [32,33]. The Perdew–Burke–Ernzerhof (PBE96) functional was used with spin polarization and dipole correction. A kinetic energy cut-off of 520 eV was used. The convergence threshold for energy was set to $10^{-6}$ eV. The conjugate gradient method was used for structural optimization with a maximum value in the interatomic forces of 0.01 eV/Å. All atomic positions were fully relaxed. Gaussian smearing with a 0.025 eV broadening was applied for the generation of total and projected density of states. The Monkhorst–Pack scheme with a *k*-centered grid was used for Brillouin zone sampling [34]. A gamma-grid of 2×2×1 was selected for the structural relaxation, and 8×8×1 was selected for the generation of the density of states. For the representation of the (111) surface of the bimetallic support, a supercell of 64 atoms was constructed (see Figure 1), maintaining at least 15 Å of vacuum in the *z*-direction. For the calculation of the d-band center, the following expression was used:

(1) $$\epsilon_{d}^{\sigma }=\frac{\int_{-\infty }^{E_{F}}\epsilon \;n_{d}^{\sigma }\left(\epsilon \right)\;d\epsilon }{\int_{-\infty }^{E_{F}}n_{d}^{\sigma }\left(\epsilon \right)\;d\epsilon }$$

where σ can take the values ↑ or ↓ for spin-up and spin-down electrons, respectively. Similarly, the occupation fraction of the *p* and *d* orbitals can be calculated from the density of states as follows:

(2) $$f_{\sigma }=\frac{\int_{-\infty }^{\epsilon_{F}}n^{\sigma }\left(\epsilon \right)\;d\epsilon }{N}$$

where *N* takes a value of 5 for *d* orbitals and 3 for *p* orbitals.

The DFT-D3 method was used to estimate the van der Waals interaction [35]. The VESTA software was utilized for the visualization of structures and density maps [36]. The analysis of the density of states and the calculation of the band center were performed using the VASPKIT code [37]. Phonon calculations were carried out using the finite-difference method (maximum ionic displacements of 0.015 Å) for ZPE + TΔS corrections to the Gibbs free energy. The electrocatalytic oxidation of glycerol (Gly) to dihydroxyacetone (DHA) proceeds through a series of key steps involving adsorption, progressive oxidation, and desorption. Initially, glycerol adsorbs onto the catalyst surface, forming the complex ${\mathrm{Gly}}^{*}$. Subsequently, the adsorbed glycerol undergoes oxidation, releasing protons ($H^{+}$) and electrons ($e^{-}$), and it transitions through intermediates such as ${G1a}^{*}$. Finally, the adsorbed dihydroxyacetone (${\mathrm{DHA}}^{*}$) desorbs from the catalyst surface, regenerating the active site for further reactions. The choice of this reaction pathway is motivated by the significance of DHA as a valuable product in the valorization of glycerol. Additionally, this pathway is particularly convenient due to its relative simplicity.

(3) $$\mathrm{Gly}+*\to {\mathrm{Gly}}^{*},$$

(4) $${\mathrm{Gly}}^{*}\to {G1a}^{*}+H^{+}+e^{-},$$

(5) $${G1a}^{*}\to {\mathrm{DHA}}^{*}+H^{+}+e^{-},$$

(6) $${\mathrm{DHA}}^{*}\to \mathrm{DHA}+*,$$

The free energies of each reaction step can be expressed as follows:

(7) $$\Delta G_{1}=G_{{\mathrm{Gly}}^{*}}-G^{*}-G_{\mathrm{Gly}},$$

(8) $$\Delta G_{2}=G_{{G1a}^{*}}-G_{{\mathrm{Gly}}^{*}}+\frac{1}{2}G_{H_{2}},$$

(9) $$\Delta G_{3}=G_{{\mathrm{DHA}}^{*}}-G_{{G1a}^{*}}+\frac{1}{2}G_{H_{2}},$$

(10) $$\Delta G_{4}=G_{\mathrm{DHA}}+G^{*}-G_{{\mathrm{DHA}}^{*}},$$

The terms $G_{{\mathrm{Gly}}^{*}}$, $G_{\mathrm{Gly}}$, $G_{{G1a}^{*}}$, $G_{{\mathrm{DHA}}^{*}}$, and $G_{\mathrm{DHA}}$ represent the Gibbs free energies of the species involved in the reaction: adsorbed glycerol, gaseous glycerol, the glycerol oxidation intermediate, adsorbed dihydroxyacetone (DHA), and gaseous DHA, respectively. These values are used to calculate the free energy changes for each reaction step.

## 3. Results

### 3.1. Influence of the Promoter on Electronic Properties

**Figure 2:**
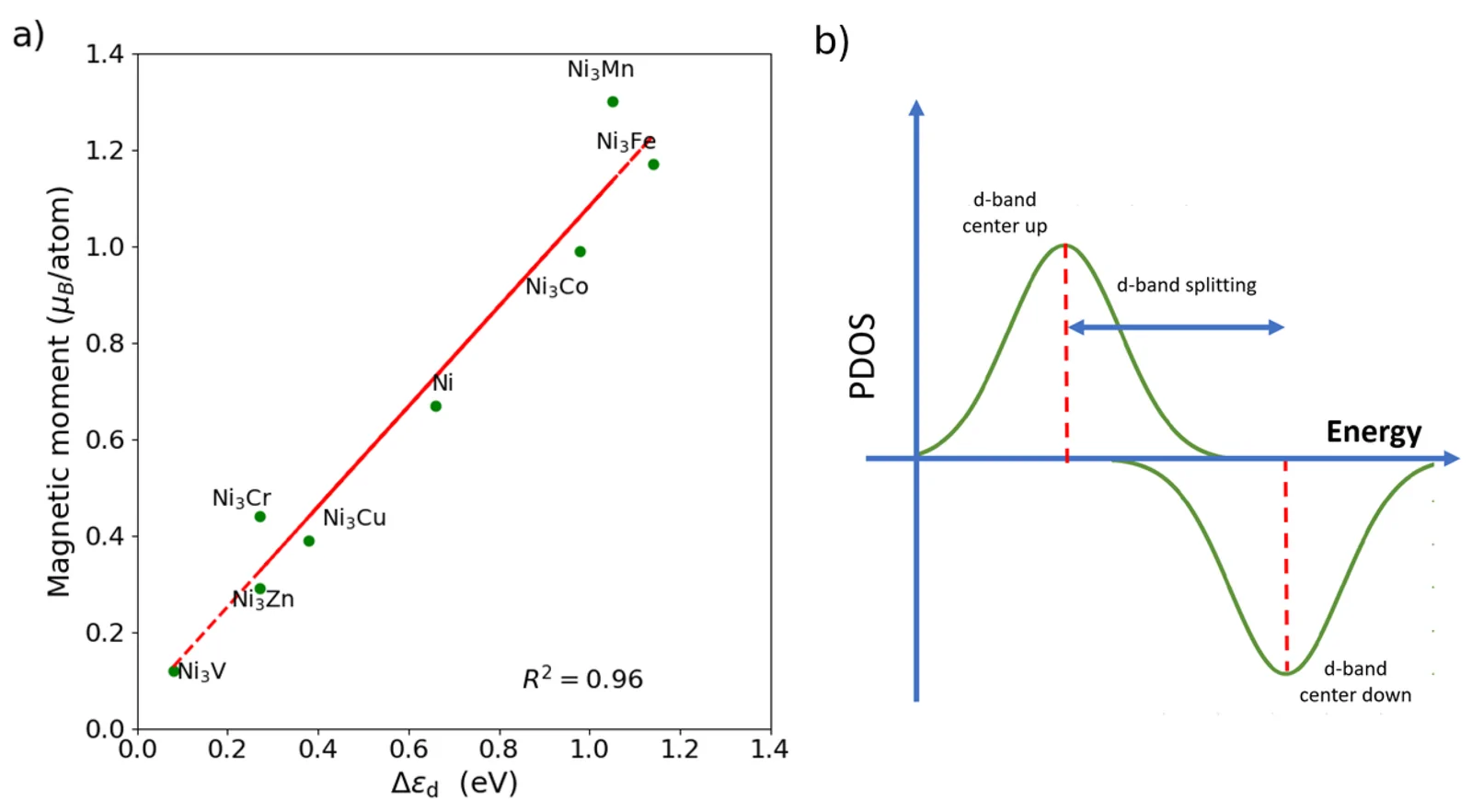
(**a**) Correlation between the magnetic moment per atom and d-band splitting for the Ni_3_X bimetallic systems under study. (**b**) Schematics depicting the concept of d-band splitting between spin-up and -down.

Figure 1a illustrates the impact of introducing a promoter on the electronic properties of the Ni (111) lattice. A strong linear correlation is observed between the magnetic moment per atom and the energy splitting of the d-band centers for spin-up and spin-down electron densities (Figure 2b). The predicted magnetic moment for the Ni (111) surface is approximately 0.67 $\mu_{B}$/atom. Some bimetallic systems, such as ${\mathrm{Ni}}_{3}$Fe, ${\mathrm{Ni}}_{3}$Co, and ${\mathrm{Ni}}_{3}$Mn, exhibit higher magnetic moment values. In contrast, systems such as ${\mathrm{Ni}}_{3}$V, ${\mathrm{Ni}}_{3}$Cr, ${\mathrm{Ni}}_{3}$Zn, and ${\mathrm{Ni}}_{3}$Cu show lower magnetic moment values than that of Ni (111).

This behavior can be attributed to differences in electronic structure and magnetic interactions among the transition metals. Elements such as Fe, Co, and Mn possess partially filled d-bands, facilitating greater spin polarization and resulting in higher magnetic moments through interaction with Ni’s d-orbitals. Conversely, metals such as Zn and Cu have nearly fully filled d-bands, contributing a few unpaired electrons, which diminishes the overall magnetic moment of the system. Additionally, while V and Cr also have partially filled d-bands, they may induce reduced spin polarization due to low band splitting, leading to lower magnetic moments in their respective bimetallic systems.

For ${\mathrm{Ni}}_{3}$V and ${\mathrm{Ni}}_{3}$Cr, the DFT calculations (Figure 2a,b) indicate a reduction in the d-band splitting between the spin-up and spin-down projected density of states (PDOS) band centers. This reduction suggests a diminished magnetic moment, which can be attributed to the electronic interactions and hybridization effects between nickel and the transition metal dopants (V and Cr). The presence of vanadium (V) and chromium (Cr), both with partially filled d-orbitals, can alter the electronic environment of the ${\mathrm{Ni}}_{3}$X system, which leads to a more significant overlap of the d-states, affecting the overall spin polarization and reducing the energy difference between the d-band centers for the two spin states.

**Figure 3:**
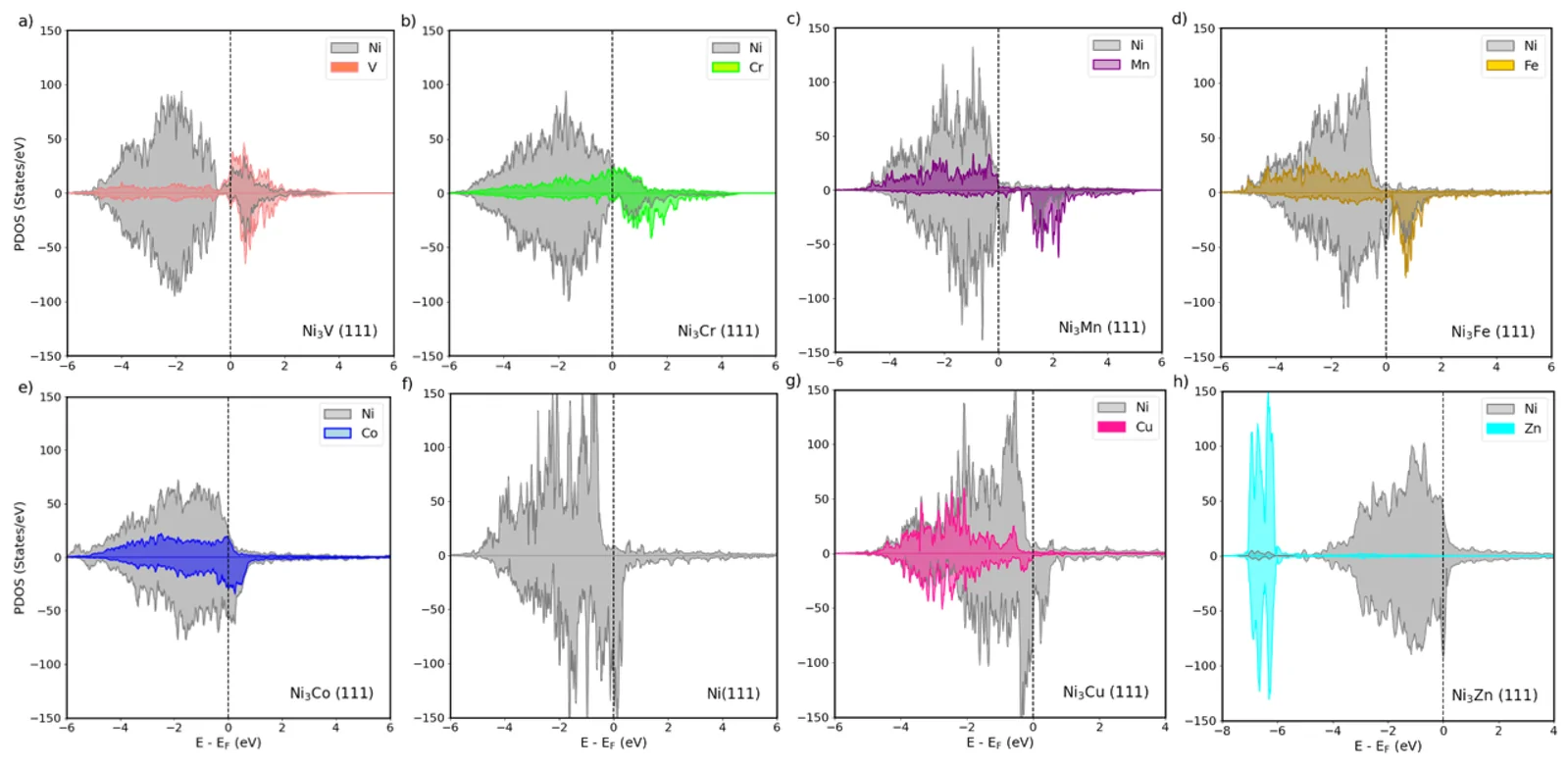
(**a**–**h**) Calculated PDOS for the Ni_3_X (111) bimetallic systems under study.

The analysis of the PDOS for various Ni-based bimetallic systems (Figure 3a–h) provides insights into their electronic and magnetic properties, reflecting the complex interplay between the nickel matrix and different promoters. In systems such as ${\mathrm{Ni}}_{3}$Fe and ${\mathrm{Ni}}_{3}$Mn (Figure 3c,d), a distinct separation in the PDOS curves for nickel and the promoter suggests strong interactions that enhance magnetic moments due to the availability of unpaired electrons in their partially filled d-orbitals. While the predicted magnetic moment for Ni (111) (PDOS shown in Figure 3f) is around 0.67 $\mu_{B}$/atom, it increases for ${\mathrm{Ni}}_{3}$Fe and ${\mathrm{Ni}}_{3}$Mn, indicating that these promoters effectively contribute to the overall magnetism through increased spin polarization.

Similarly, for ${\mathrm{Ni}}_{3}$Cu and ${\mathrm{Ni}}_{3}$Zn (Figure 3g,h), the PDOS indicates that the promoters possess full d-orbitals, significantly limiting their contribution to the magnetic moment of the system. This factor is crucial for understanding why these bimetallic systems demonstrate lower magnetic properties than their ${\mathrm{Ni}}_{3}$Fe and ${\mathrm{Ni}}_{3}$Mn counterparts. The overlap of the PDOS for Ni and its promoters in these systems further emphasizes the loss of magnetic interaction, as the nearly complete d-bands do not allow for significant spin polarization.

Overall, the findings underscore the critical role of the electronic configuration of the promoters in determining the magnetic characteristics of Ni-based bimetallic compounds. Elements such as Mn and Fe enhance magnetism by providing unpaired electrons that are conducive to spin alignment, whereas V, Cr, Cu, and Zn, with their electronic structures, lead to a diminished magnetic response. This comprehensive understanding of the PDOS across different bimetallic systems illuminates the intricate relationships between composition, electronic structure, and magnetic properties, providing valuable insights for designing materials with tailored magnetic characteristics.

### 3.2. Influence on the Adsorption of Glycerol Molecules

**Figure 4:**
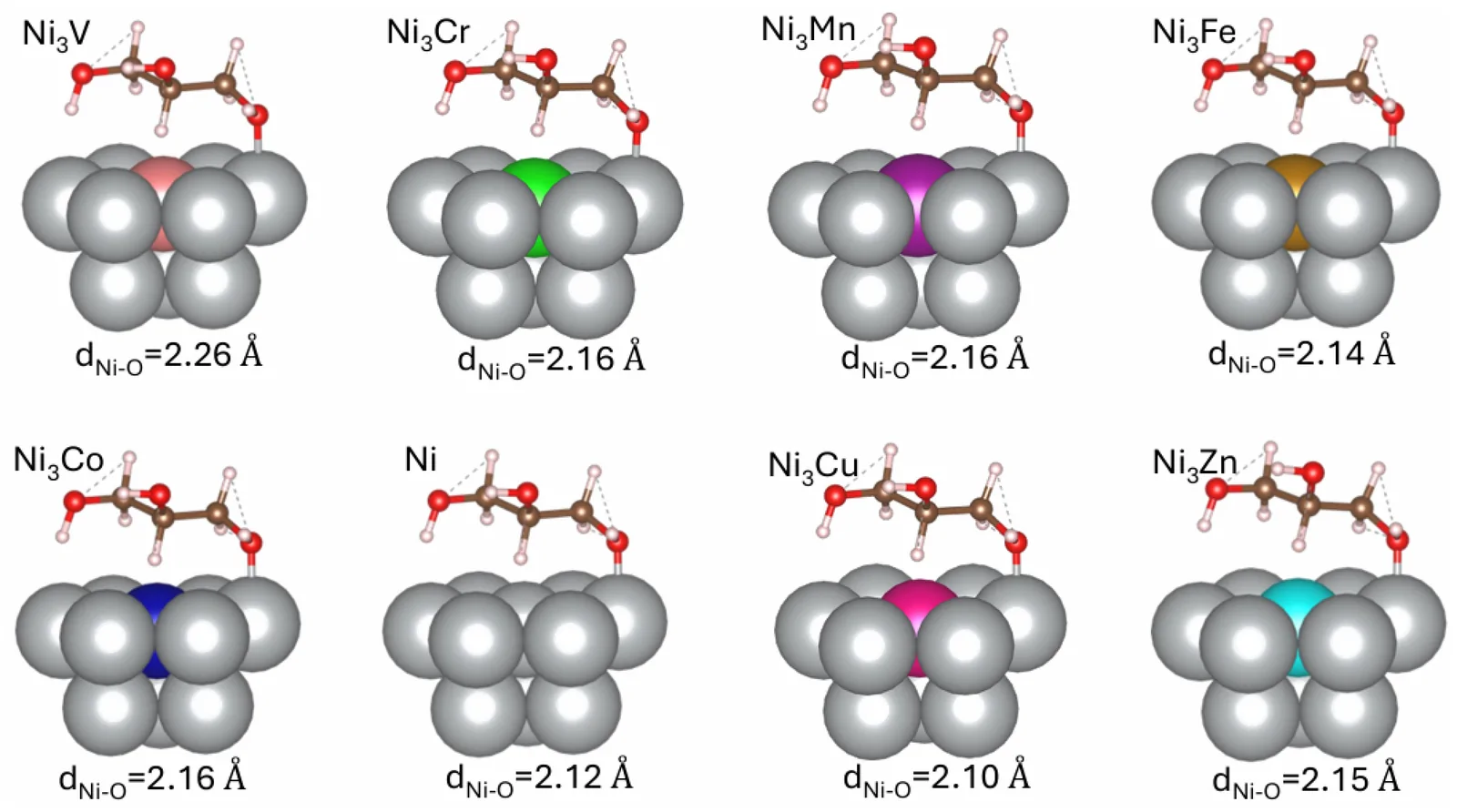
Atomic structure for a glycerol molecule adsorbed on Ni_3_X (111) surfaces.

Figure 4 shows the optimized adsorption structures of a glycerol molecule on the surfaces of Ni-based bimetallic systems. In these models, oxygen atoms (represented as red spheres) are adsorbed onto the surfaces of nickel atoms (gray spheres), and the Ni-O bond distances are labeled for each system. The Ni-O bond lengths vary from 2.10 Å in ${\mathrm{Ni}}_{3}$Cu to 2.26 Å in ${\mathrm{Ni}}_{3}$V, with pure Ni showing a bond length of 2.12 Å. Each bimetallic system includes a different transition metal, depicted in distinct colors: vanadium (V, pink), chromium (Cr, green), manganese (Mn, purple), iron (Fe, gold), cobalt (Co, blue), copper (Cu, magenta), and zinc (Zn, cyan).

These optimized structures highlight the differences in Ni-O bonding across the various alloys, with oxygen consistently adsorbing onto Ni atoms rather than the promoter atoms. Qualitatively, this preference for Ni could be attributed to nickel’s partially filled d-band, which requires two electrons for full filling according to the ten-electron rule. Nickel can readily accept electron density, and the lone pair of electrons on the oxygen atom in the OH group of glycerol can donate this electron density. This interaction fulfills Ni’s electronic requirement for additional electrons, stabilizing its configuration on the surface.

**Figure 5:**
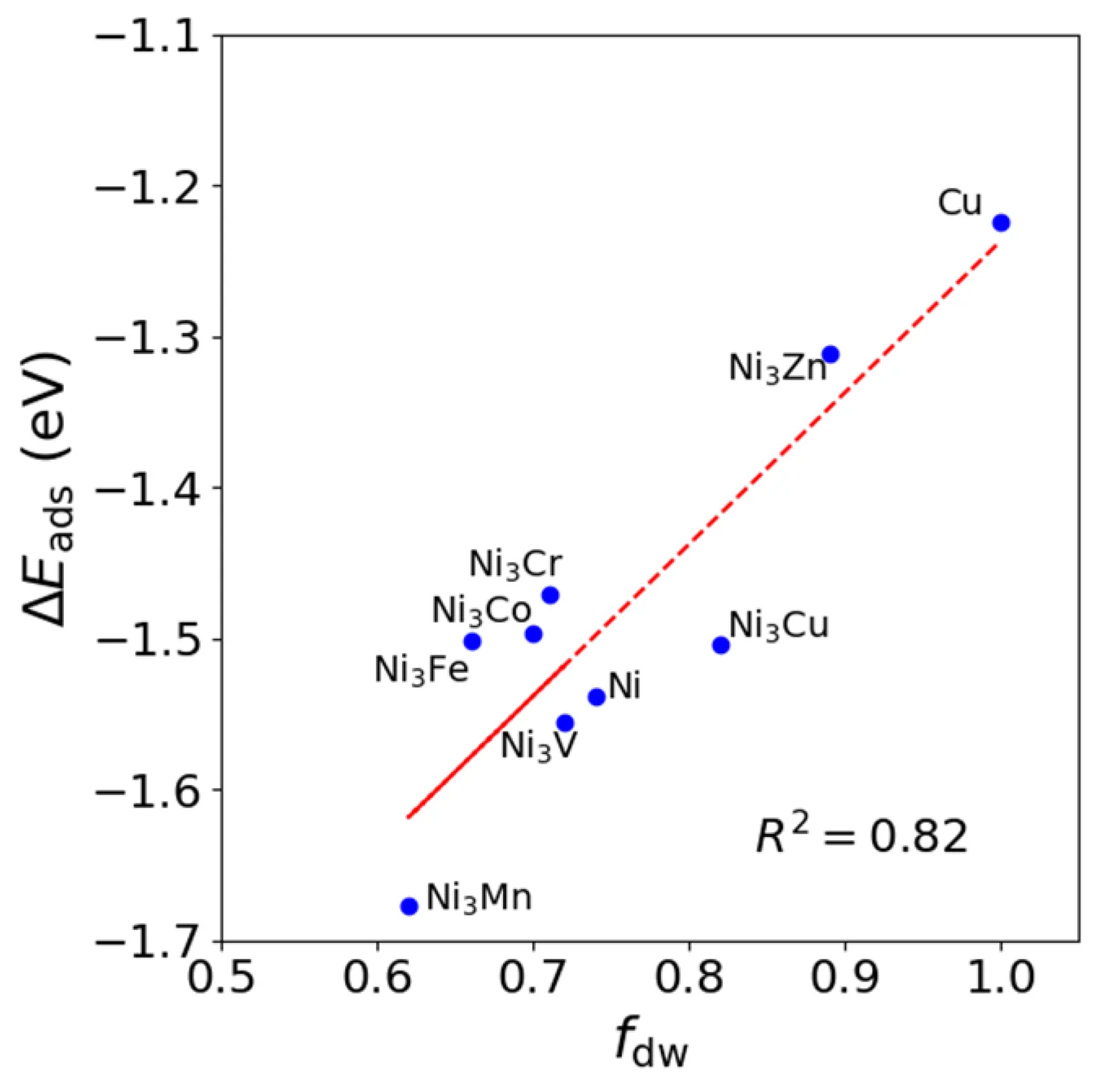
Correlation between the adsorption energy and d-band filling factor $f_{\mathrm{dw}}$ of Ni_3_X (111) surfaces.

Figure 5 illustrates the relationship between the adsorption energy $E_{\mathrm{ads}}$ of glycerol in electron volts (eV) and the d-band occupancy fraction for the spin-down PDOS ($f_{\mathrm{dw}}$) for various transition metal compounds and elements. The y-axis represents the adsorption energy, ranging from approximately −1.1 eV to −1.7 eV, while the x-axis indicates the d-band occupancy fraction, spanning from 0.5 to 1.0. A red dashed line indicates the trend, suggesting a negative correlation between the d-band occupancy fraction and adsorption energy. The correlation coefficient $R^{2}=0.82$ reinforces this, showing that as the d-band occupancy increases, the adsorption energy becomes more favorable (i.e., more negative).

Additionally, the implications of this correlation suggest that a lower d-band occupancy indicates that systems with higher spin polarization exhibit more negative adsorption energy.

### 3.3. d-Band Model Analysis

The d-band model is illustrative in elucidating results, effectively demonstrating how variations in d-band occupancy and band centers impact the adsorption energy. Recently, Saini et al. highlighted the relationship between occupancies and the contribution of d-orbitals to adsorption energy ($\Delta E_{d}^{A}$) through a two-site Hamiltonian model [23]:

(11) $$\Delta E_{d}^{A}≃-\left(1-f_{-}\right)\frac{V^{2}}{|\epsilon_{d}-\epsilon_{a}|}+\left(1+f_{-}\right)a_{s}V^{2}+\left(f_{-}-f_{d}^{\mathrm{site}}\right)\epsilon_{d}$$

where $\epsilon_{d}$ and $\epsilon_{a}$ are the energies of the d-band and adsorbate, respectively, before the adsorption. $f_{-}$ and $f_{d}^{\mathrm{site}}$ represent the occupancies of the antibonding orbital and metal site after adsorption, *V* is a coupling matrix element between adsorbate and metal states, and $a_{s}$ is a constant relating the coupling matrix *V* with the overlap *S* between adsorbate and metal d-orbitals.

**Figure 6:**
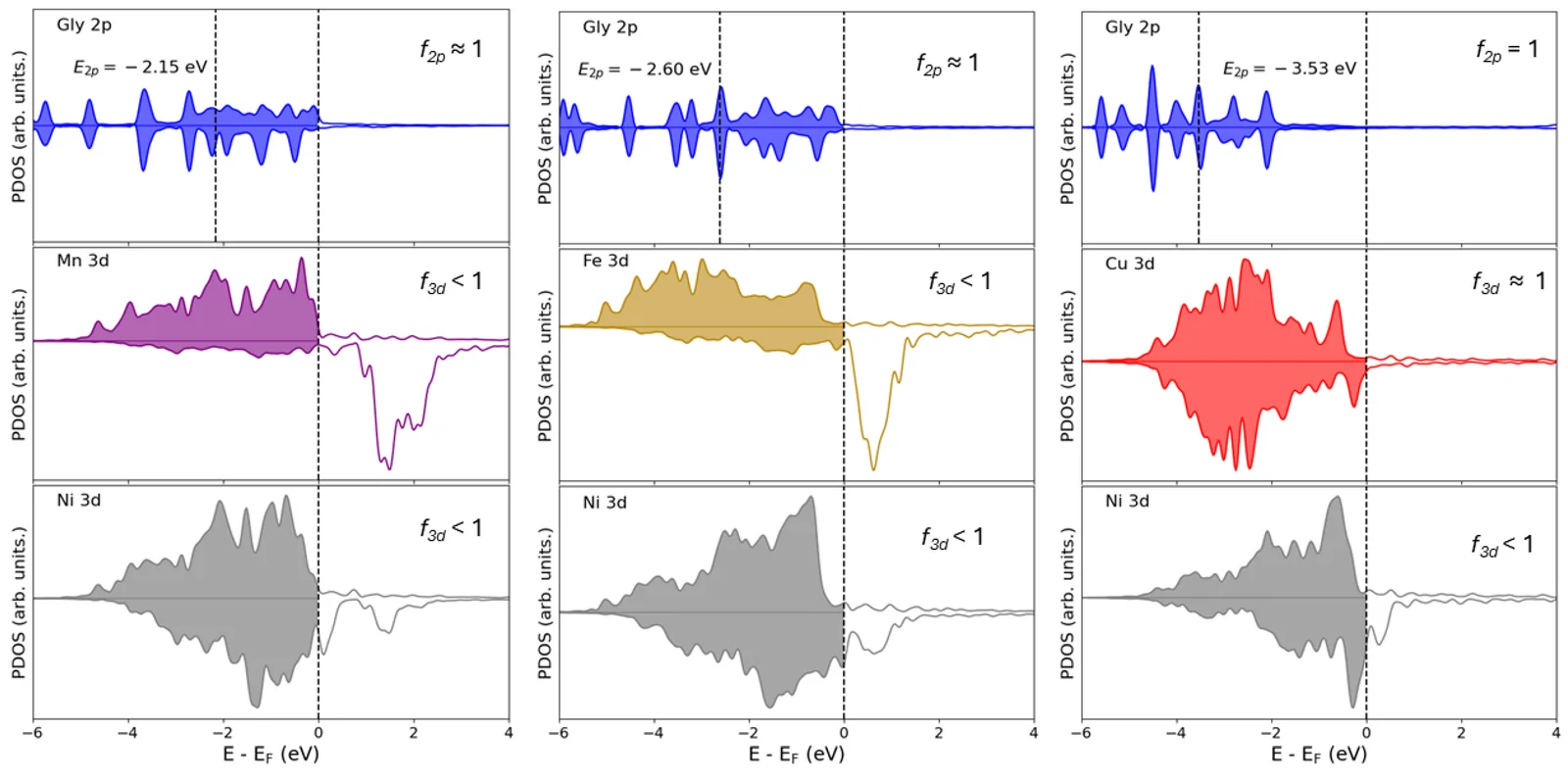
Comparison of the PDOS for a glycerol molecule adsorbed on Ni_3_Mn, Ni_3_Fe, and Ni_3_Cu surfaces. The labels show the different band filling for metals ($f_{d}$) and adsorbate ($f_{2p}$).

Beyond its predictive capacity, which has been discussed by various authors, including systematic improvements [31], the true utility of Equation (10) lies in its ability to visualize the electronic factors that most influence the reactivity of metal surfaces. Figure 6 illustrates how Equation (10) can be used to analyze the reactivity of a surface in terms of the PDOS.

Figure 6 illustrates the PDOS for the 2*p* orbitals of the OH group in glycerol interacting with three different bimetallic surfaces: ${\mathrm{Ni}}_{3}$Mn, ${\mathrm{Ni}}_{3}$Fe, and ${\mathrm{Ni}}_{3}$Cu. In each column, the top panel shows the PDOS of the 2*p* orbitals of the OH group, while the middle and bottom panels represent the PDOS of the 3*d* orbitals for the respective metals in the bimetallic systems.

For the OH group, the PDOS reveals peaks indicating the energies at which the 2*p* orbitals of the OH group interact with the metal surface. For each system, the energy of the OH group’s 2*p* orbital shifts depending on the electronic structure of the bimetallic compound. Specifically, the 2*p* band center of OH appears at −2.15 eV for ${\mathrm{Ni}}_{3}$Mn, −2.60 eV for ${\mathrm{Ni}}_{3}$Fe, and −3.53 eV for ${\mathrm{Ni}}_{3}$Cu, reflecting how each metal influences the electronic interaction with the adsorbate.

The 3*d* orbitals of the metals also exhibit differences in their electronic structure, with partial occupancy ($f_{\mathrm{dw}}<1$) for Mn, Fe, and Ni and a fully occupied d-band ($f_{\mathrm{dw}}=1$) for Cu, thus influencing the adsorption strength.

The energies and occupations of both the d orbitals of the bimetallic surfaces and the p orbitals of the OH group in glycerol play a critical role in determining the adsorption strength. In ${\mathrm{Ni}}_{3}$Mn and ${\mathrm{Ni}}_{3}$Fe, the d-band is only partially filled, as indicated by the occupation fraction $f_{\mathrm{dw}}<1$. This partial filling allows for stronger hybridization between the 2*p* orbitals of the OH group and the 3*d* orbitals of the metal, leading to significant interaction, as seen with the higher energy of the 2*p* orbitals closer to the Fermi level. This higher energy correlates with a stronger adsorption strength, as a more significant overlap between the metal d-states and the OH p-states facilitates better orbital mixing and binding.

In contrast, in ${\mathrm{Ni}}_{3}$Cu, where the 3*d* band is fully occupied ($f_{\mathrm{dw}}=1$), the OH 2*p* orbital is much lower in energy (−3.53 eV), indicating weaker interaction. Since the Cu d-band is filled, it lacks available states to hybridize with the OH group, resulting in a weaker bond strength and lower adsorption energy. To complement the description, Table 1 presents the electronic occupations of the 3d orbital ($f_{\mathrm{up}}/f_{\mathrm{dw}}$) and the O-2p orbital ($f_{\mathrm{up}}/f_{\mathrm{dw}}$) for all materials under study with an adsorbate. The values for the 3d orbital range from 0.76/0.62 to 1.00/1.00, while those for the O-2p orbital vary between 0.93/0.95 and 1.00/1.00, reflecting the differences in electronic distribution and the interactions between the metallic species and the adsorbate.

Overall, systems with higher d-band occupancy (${\mathrm{Ni}}_{3}$Cu) exhibit weaker adsorption due to the reduced availability of unoccupied d-states to interact with the adsorbate. Conversely, lower d-band occupancy (as in ${\mathrm{Ni}}_{3}$Mn and ${\mathrm{Ni}}_{3}$Fe) enables more efficient orbital hybridization, leading to stronger adsorption.

The correlation between the position of the 2*p* orbital of OH and the d-band occupation emphasizes the importance of the electronic structure of the adsorbate in determining the adsorption energy; a lower 2*p* energy with respect to $E_{F}$ indicates stronger metal–adsorbate interaction, while a higher 2*p* energy corresponds to weaker bonding.

### 3.4. Intermediates of the Reaction

**Figure 7:**
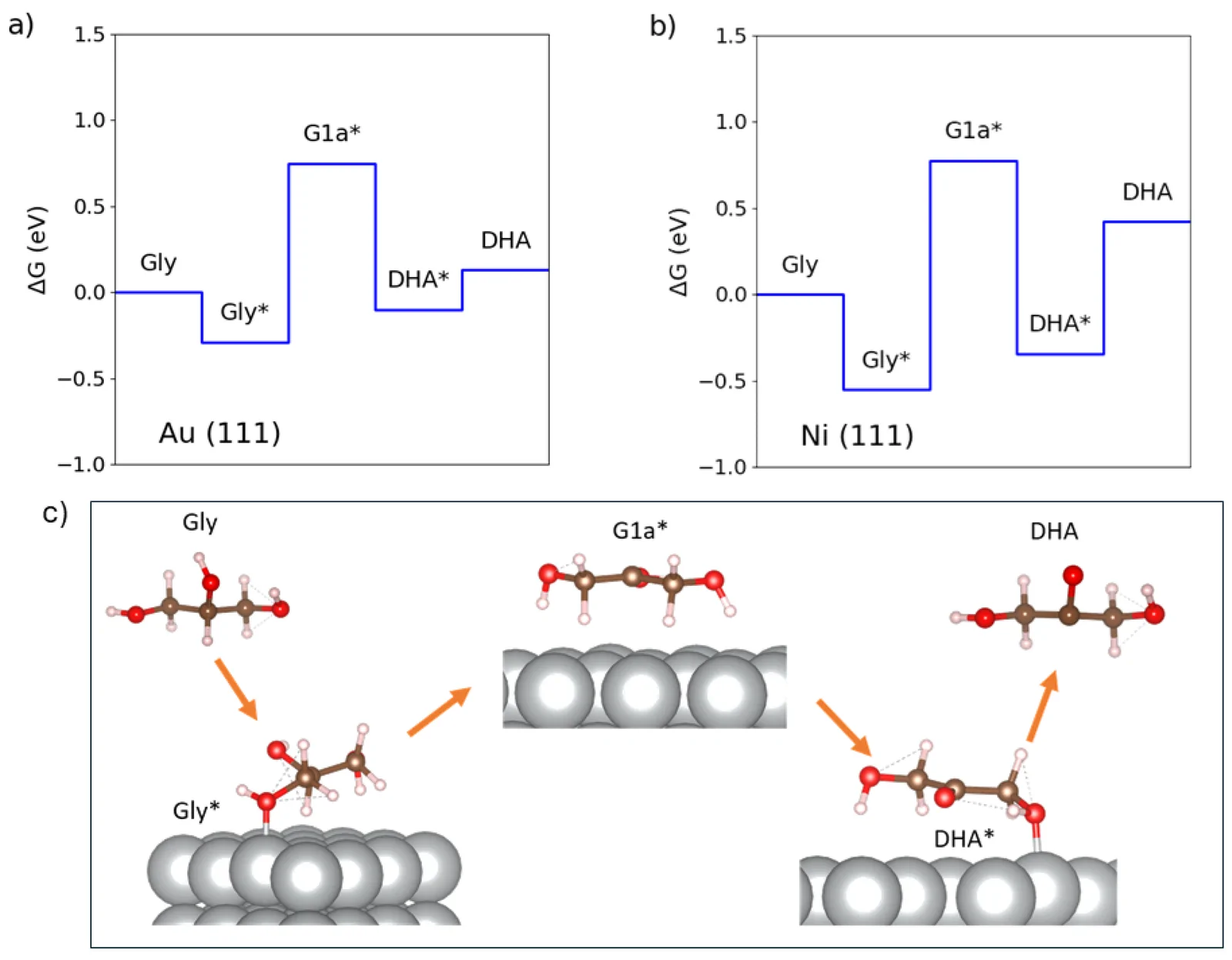
(**a**) Calculated free energy diagram for the electro-oxidation of glycerol to DHA for a Au (111) surface and (**b**) for a Ni (111) surface, and (**c**) illustrates the structural configurations of the intermediates on Ni (111).

The electro-oxidation of glycerol (GLY) can proceed via different reaction pathways, yielding a range of products, such as glyceraldehyde, glyceric acid, and dihydroxyacetone (DHA), among others [38,39]. To better understand these processes, the production of DHA has emerged as a key pathway in the Au-catalyzed electro-oxidation of glycerol (EOG). The oxidation primarily occurs at the secondary C group of glycerol, resulting in DHA as the major product [40,41,42]. For this reason, it is interesting to compare the results. Figure 7 presents a comparative analysis of the energy profiles and structural intermediates involved in the electrooxidation of glycerol (Gly) to dihydroxyacetone (DHA) on Au (111) and Ni (111) surfaces. In panels (a) and (b), the Gibbs free energy (ΔG) diagrams highlight the reaction pathway, showing the adsorption of glycerol (Gly → Gly*), the formation of an intermediate (G1a*), and the final product (DHA*). The energy profiles reveal that while glycerol adsorption is more exergonic on Ni (111), suggesting stronger interactions, the transition from the DHA* intermediate to the final product is more energetically favorable on Au (111). This indicates that Au (111) facilitates the release of DHA more efficiently than Ni (111) due to the lower energy barrier for this final step. Panel (c) illustrates the structural configurations of the intermediates on Ni (111), where glycerol and its oxidized intermediates are adsorbed via oxygen atoms, indicating strong metal–oxygen interactions. However, despite Ni (111)’s strong initial adsorption, Au (111) proves more effective for the final DHA desorption.

**Figure 8:**
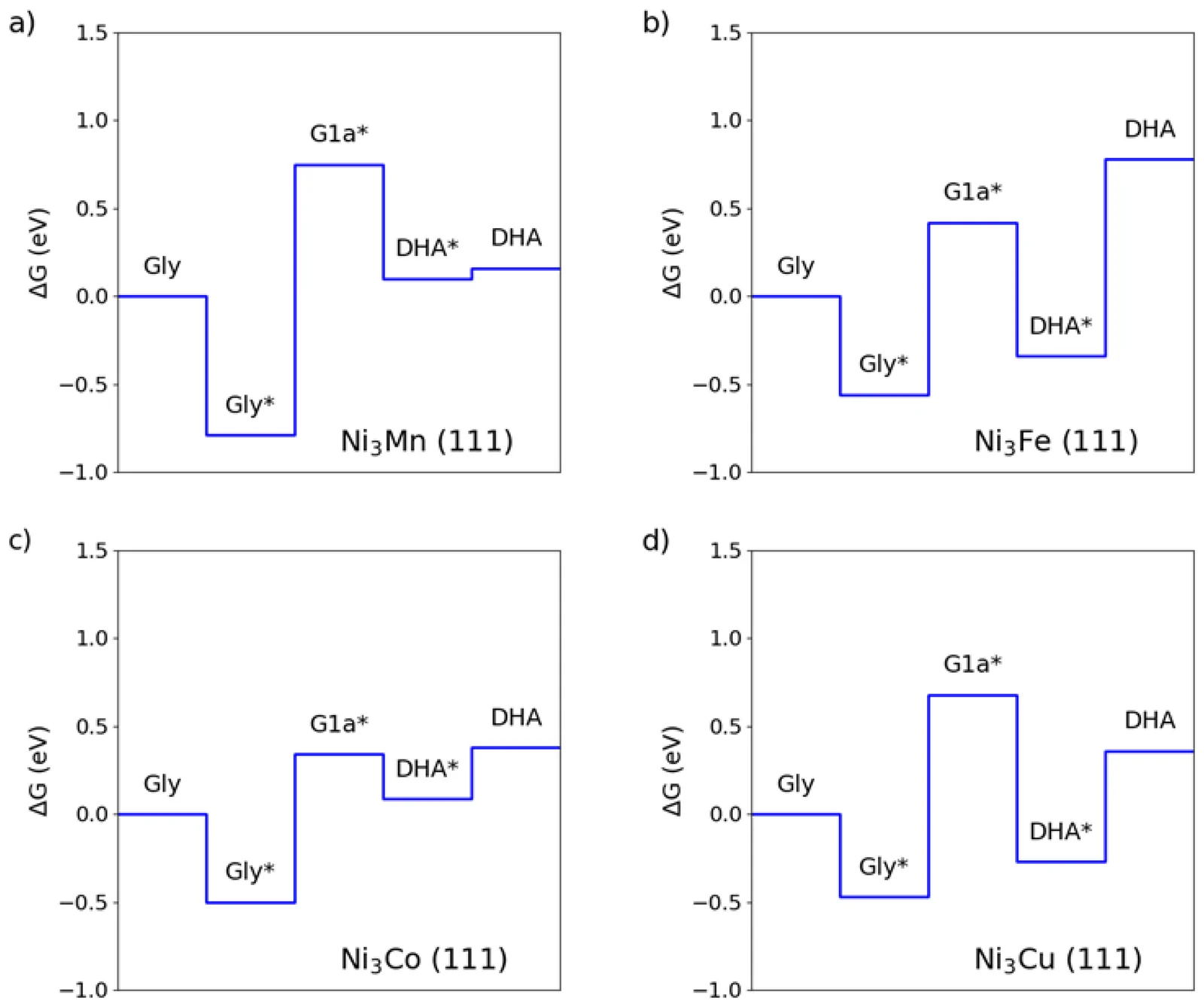
(**a**–**d**) Calculated free energy diagram for the electro-oxidation of glycerol to DHA on Ni_3_Mn, Ni_3_Fe, Ni_3_Co, and Ni_3_Cu (111) surfaces.

**Figure 9:**
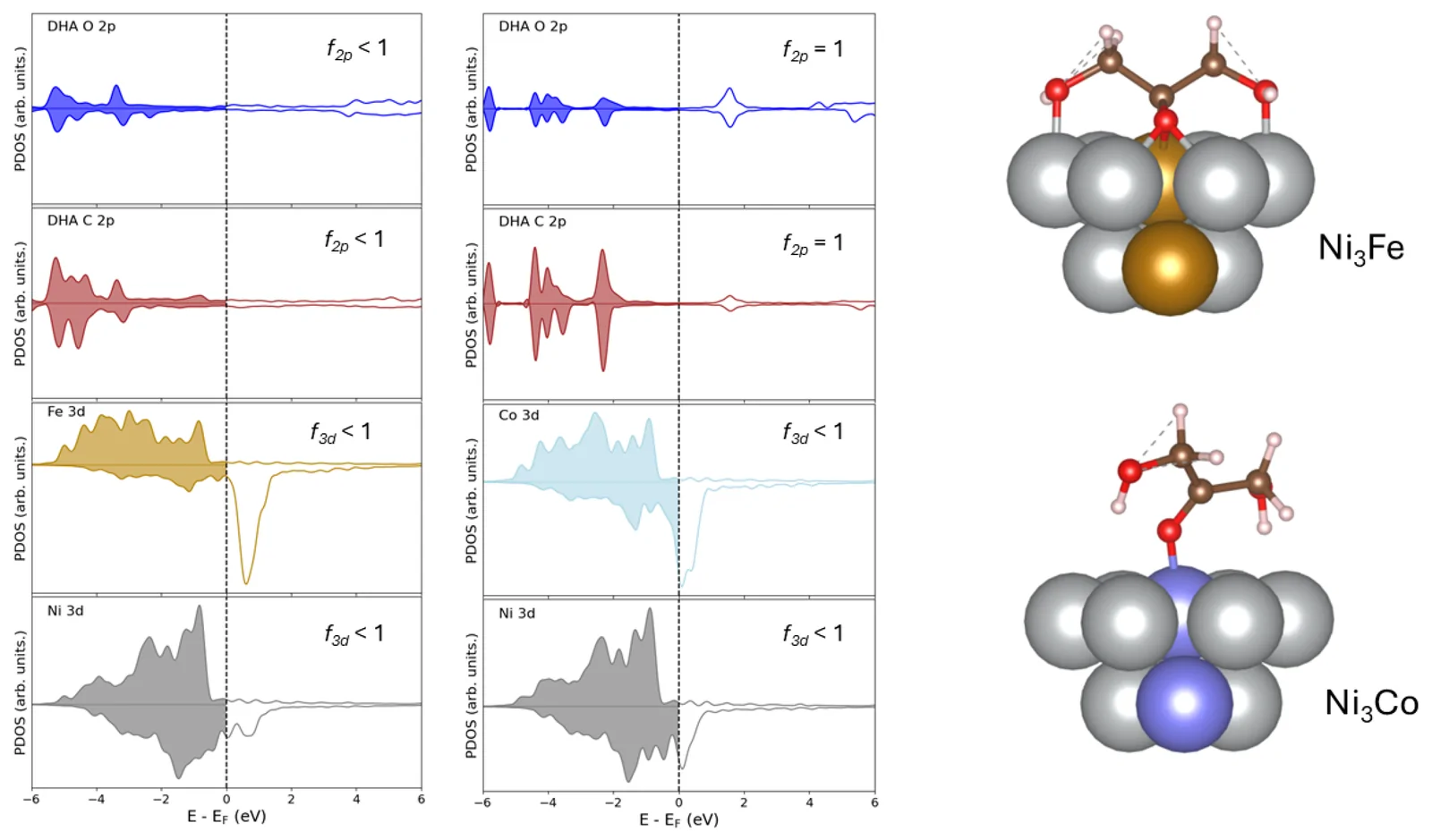
Comparison of PDOS for the 2p orbitals of oxygen and carbon in a DHA molecule adsorbed on ${\mathrm{Ni}}_{3}$Co and ${\mathrm{Ni}}_{3}$Fe surfaces (first and second panels).

Figure 8 presents the Gibbs free energy (ΔG) diagrams for the electro-oxidation of glycerol (Gly) to DHA on various ${\mathrm{Ni}}_{3}$X (111) alloy surfaces, where X represents Mn, Fe, Co, and Cu. The diagrams depict the energy progression for key steps: glycerol adsorption (Gly → Gly*), intermediate formation (G1a*), and DHA adsorption and release (DHA* → DHA). On ${\mathrm{Ni}}_{3}$Mn, glycerol adsorption is highly exergonic, with a significant energy drop, followed by a sharp increase during G1a* formation and a moderate barrier for DHA release. For ${\mathrm{Ni}}_{3}$Fe, glycerol adsorption is relatively moderate, but G1a* formation is the most energy-demanding step across all alloys, and DHA release is hindered by a considerable energy barrier. In the case of ${\mathrm{Ni}}_{3}$Co, glycerol adsorption is similar to that of ${\mathrm{Ni}}_{3}$Mn, and although G1a* formation remains energy-costly, the final DHA release occurs with relatively low energy expenditure, indicating a more efficient process. ${\mathrm{Ni}}_{3}$Cu shows moderately exergonic glycerol adsorption, with a Gibbs free energy profile similar to that of ${\mathrm{Ni}}_{3}$Co, and while G1a* formation follows the same trends as other surfaces, DHA release is particularly favorable. Thus, ${\mathrm{Ni}}_{3}$Co and ${\mathrm{Ni}}_{3}$Cu (111) stand out as promising candidates for the electro-oxidation of glycerol to DHA due to their moderate glycerol adsorption and favorable energy profiles for DHA release, while ${\mathrm{Ni}}_{3}$Fe is the least efficient due to the high energy required for DHA release. At this point, it is particularly interesting to explore the electronic factors responsible for ${\mathrm{Ni}}_{3}$Fe’s strong interaction with DHA. Figure 9 presents the projected density of states (PDOS) for the 2p orbitals of oxygen and carbon in the DHA molecule adsorbed on ${\mathrm{Ni}}_{3}$Co and ${\mathrm{Ni}}_{3}$Fe surfaces, as well as for the 3d orbitals of the metal atoms (Ni, Co, and Fe). Notably, in the case of ${\mathrm{Ni}}_{3}$Fe, the occupation factors for both the 2p and 3d orbitals are less than 1, indicating partial filling of both adsorbate and surface states. In contrast, for DHA adsorbed on the ${\mathrm{Ni}}_{3}$Co system, the 2p states of O and C in the DHA molecule show occupation factors of 1, indicating full filling of the adsorbate states around the Fermi energy, while the partially filled 3d orbitals of Ni and Co remain spin-polarized. Based on this comparison, we can confirm that not only does the position of the d-band relative to the Fermi level define the interaction energy, but the position and occupancy of the adsorbed molecule’s states also play a crucial role. Specifically, an occupancy of less than 1 is associated with a higher interaction energy, as qualitatively predicted by the d-band model presented in Equation (10).

## 4. Conclusions

This study highlights the critical influence of promoters on the electronic and magnetic properties of Ni-based bimetallic systems, as well as on the adsorption and reactivity of the glycerol molecule. A strong correlation was observed between the magnetic moment and d-band splitting across various systems, where elements such as Fe and Mn significantly enhanced magnetism by providing unpaired electrons, while metals with filled d-bands, such as Cu and Zn, diminished this property.

Moreover, glycerol’s adsorption on ${\mathrm{Ni}}_{3}$X surfaces demonstrated that lower d-band occupancy is associated with higher adsorption energy due to increased orbital hybridization between the metals and the OH groups in glycerol. In particular, the interaction with dihydroxyacetone (DHA) was found to depend strongly on the nature of the metals in the ${\mathrm{Ni}}_{3}$X catalysts. For instance, ${\mathrm{Ni}}_{3}$Fe, which has partially filled d-bands, exhibited stronger binding with DHA due to enhanced orbital overlap with the partially filled 2*p* orbitals of DHA. This interaction illustrates how the presence of unfilled orbitals in the adsorbate can lead to increased reactivity and stabilization of the adsorbed intermediate, demonstrating that DHA on ${\mathrm{Ni}}_{3}$Fe serves as a prime example of how partial band filling enhances interactions.

Conversely, in the case of ${\mathrm{Ni}}_{3}$Co, where the d-bands are filled, such favorable orbital overlap does not occur, resulting in weaker binding and reactivity with DHA. Furthermore, when considering glycerol’s electro-oxidation, the energy diagrams for ${\mathrm{Ni}}_{3}$Co and ${\mathrm{Ni}}_{3}$Cu reveal these systems to be interesting candidates due to their favorable characteristics in the catalytic process.

These findings provide detailed insights into how promoters and electronic structures affect both magnetic properties and surface reactivity. The results offer valuable guidelines for designing materials with optimized characteristics for catalytic applications, emphasizing the role of electronic and magnetic properties in determining the performance of bimetallic systems.

## Figures and Tables

**Table 1:** The 3d and O-2p occupations with an adsorbate.

Species	3d Occupation ($f_{\mathrm{up}}/f_{\mathrm{dw}}$)	O-2p Occupation ($f_{\mathrm{up}}/f_{\mathrm{dw}}$)
Ni_3_Cr	0.76/0.73	0.95/0.96
Ni_3_V	0.79/0.74	0.94/0.95
Ni_3_Mn	0.88/0.62	0.93/0.95
Ni_3_Fe	0.90/0.68	0.96/0.94
Ni_3_Co	0.93/0.72	0.97/0.97
Ni	0.92/0.78	0.98/0.96
Ni_3_Cu	0.94/0.87	1.00/1.00
Ni_3_Zn	0.95/0.90	1.00/1.00
Cu	1.00/1.00	1.00/1.00

## Data Availability

The original contributions presented in this study are included in the article. Further inquiries can be directed to the corresponding authors.
